# Cardiac angiosarcoma: how multimodality imaging and excimer laser assisted intracardiac biopsy led to the correct diagnosis—a case report

**DOI:** 10.1093/ehjcr/ytae433

**Published:** 2024-08-24

**Authors:** Anna Veliqi, Samer Hakmi, Lukas Kaiser, Stephan Willems, Da-Un Chung

**Affiliations:** Department of Cardiology & Critical Care Medicine, Asklepios Klinik St. Georg, Lohmühlenstraße 5, 20099 Hamburg, Germany; Department of Cardiac Surgery, Asklepios Klinik St. Georg, Lohmühlenstraße 5, 20099 Hamburg, Germany; Department of Cardiology & Critical Care Medicine, Asklepios Klinik St. Georg, Lohmühlenstraße 5, 20099 Hamburg, Germany; Department of Cardiology & Critical Care Medicine, Asklepios Klinik St. Georg, Lohmühlenstraße 5, 20099 Hamburg, Germany; Department of Cardiology & Critical Care Medicine, Asklepios Klinik St. Georg, Lohmühlenstraße 5, 20099 Hamburg, Germany

**Keywords:** Cardiac imaging, Echocardiography, Cardiac tumours, Excimer laser, Transvenous lead extraction, Case report

## Abstract

**Background:**

Cardiac angiosarcomas are exceptionally uncommon, and result in significant morbidity and mortality. Utilizing a multimodality approach enhances the characterization of the mass for optimal diagnostic outcomes. The recommended primary treatment involves complete surgical resection coupled with adjuvant radiochemotherapy. Excimer laser sheaths provide a novel option for extracting substantial tissue samples, facilitating appropriate and targeted treatment.

**Case summary:**

A 54-year-old female presented with dyspnoea and chest pain. Cardiac imaging showed a large right atrial mass suspected to be malignant. Echocardiography was utilized for diagnosis, follow-ups, and as part of the biopsy procedure. However, adopting an off-label approach involving an excimer laser sheath as a bioptome led to the successful diagnosis of an angiosarcoma. Commencement of radiochemotherapy resulted in a reduction in tumour size and a rapid improvement in the patient’s quality of life.

**Discussion:**

The presented case underlines the significance of multimodality imaging and the use of an excimer laser-assisted intracardiac biopsy in achieving an accurate diagnosis. Cardiovascular imaging not only serves as the primary diagnostic tool but also plays a crucial role in risk stratification and in planning therapeutic interventions.

**Learning points**

- Identifying a cardiac tumour can be quite challenging, but recent advances of cardiovascular imaging empower clinicians to refine potential diagnoses before resorting to invasive biopsies.
- A multimodality approach seems to enhance the characterization of a mass for optimal results.

## Introduction

Primary cardiac tumours are exceedingly rare: according to autopsy studies, their prevalence is only ∼0.02%. The angiosarcoma accounts for 30% of malignant primary tumours of the heart, which predominantly presents in the right atrium, but can occur in any cardiac chamber.^1^ The prognosis is mostly poor due to inoperability at the time of diagnosis. Without treatment, life expectancy is described to be less than one year.^2^ Correct diagnosis and treatment rely on modern multimodality imaging with echocardiography as its mainstay and cardiac computed tomography (CT) and magnetic resonance imaging (MRI) as complementary adjuncts for spatial resolution and tissue differentiation.^3^ The recommended therapy of choice is complete surgical resection combined with adjuvant radiochemotherapy. Due to its rarity, there are no large controlled studies on angiosarcoma treatment.^4^ Anthracycline-based chemotherapy combined with stereotactic radiation led to a high incidence of therapy-related cardiotoxicity. Various case reports have described alternative regimens with paclitaxel combined with radiotherapy with more encouraging results.^5^

## Summary figure

**Figure:**
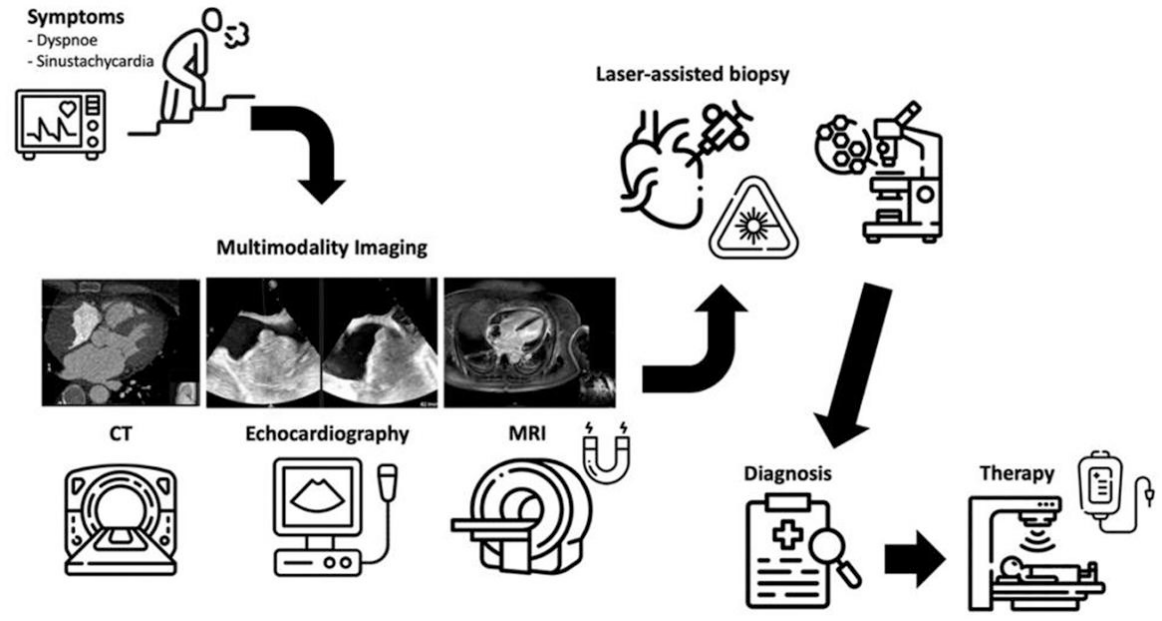


## Case presentation

**Figure 1:**
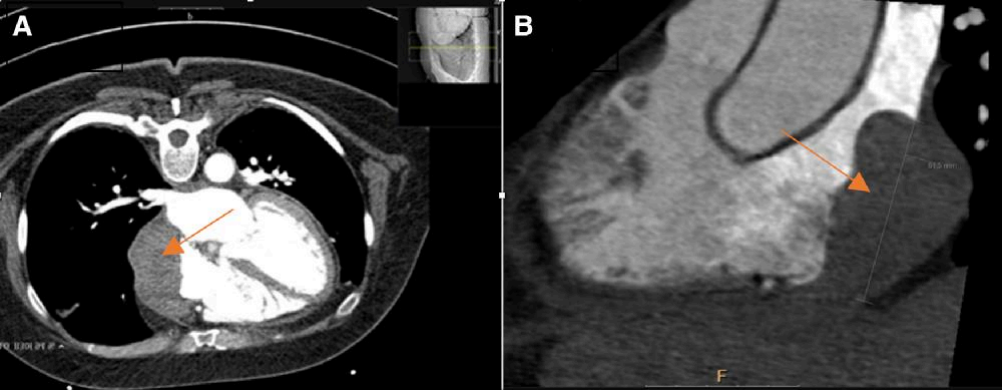
CT scan in axial layering (arterial phase) showing a smooth-bordered, inhomogeneously enhancing lesion of the wall of the right atrium (diffuse intramural growth). (*A*) CT scan in parasagittal view demonstrating the lesion, measuring up to a maximum of 6.2 cm, bulging convexly into the pulmonary trunk (*B*).

**Figure 2:**
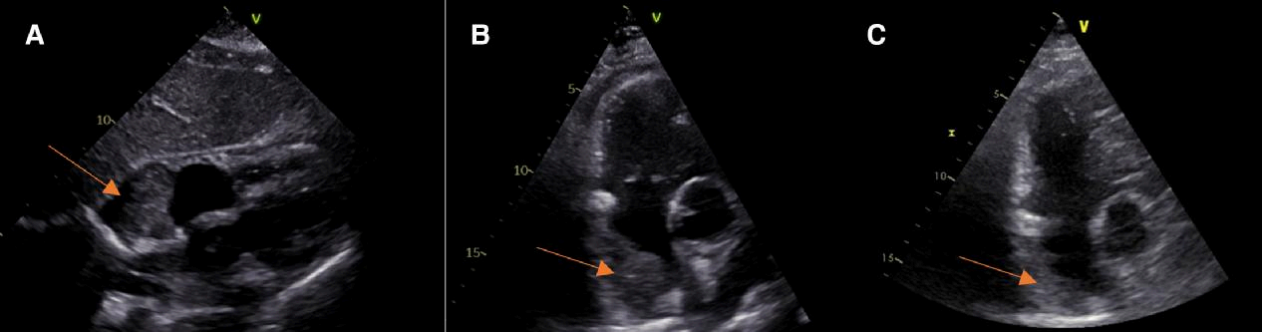
Transthoracic echocardiogram with subcostal plane (*A*) and short axis view (*B*) demonstrating the large right atrial mass (arrows) in the area of the right atrium with inhomogenous tissue contrast and smooth borders. (*C*) Showing the size regression of the mass in follow-up in short axis view.

**Figure 3:**
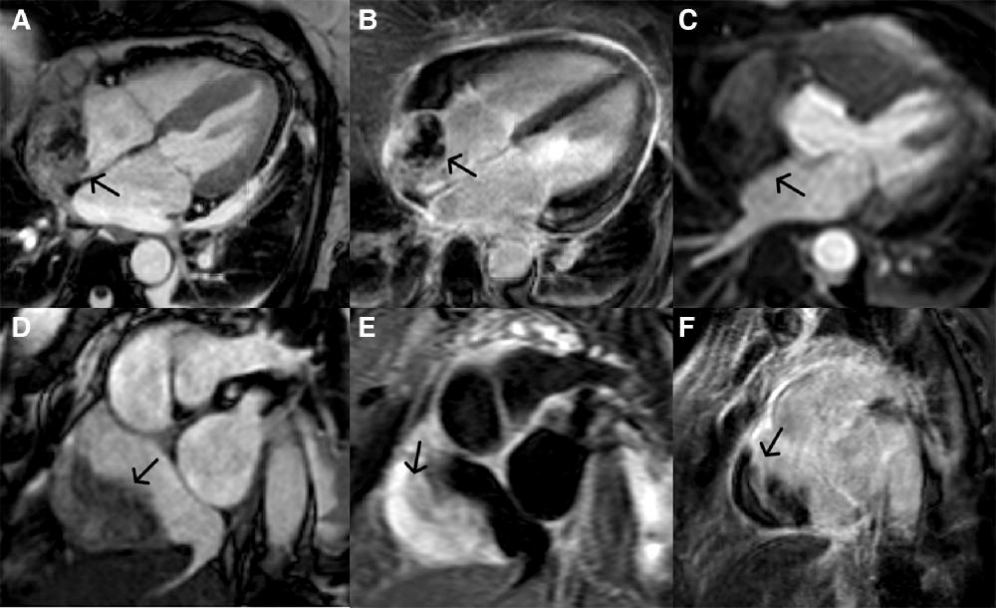
Cardiac MRI demonstrating a mass in the wall of the right atrium with suspected pericardial infiltration in cine imaging (bFFE; four-chamber view). (*A*) The following late enhancement sequence (SPIR; four-chamber view) is showing peripherally accentuated, heterogeneous late enhancement in the lesion. Furthermore contrast enhancement (gadolinium) of the pericardium can be observed, as well as subepicardial enhancement (midventricular lateral). (*B*) Perfusion images, showing no contrast enhancement in the mass and remaining myocardium. (*C*) Short axis view in cine imaging (bFFE) (*D*), T2W-STIR imaging (showing a hyperintense lesion) (*E*) and LGE imaging for further characterization of the mass in different views (*F*).

We are reporting a case of a 54-year-old female patient, who presented to the emergency department with exertional dyspnoea (NYHA class III) and chest pain. She was otherwise in good general condition and exhibited no other symptoms such as fever, malaise, or weight loss. Her past medical history was unremarkable with arterial hypertension. On clinical examination, no abnormalities were observed. An electrocardiogram showed sinustachycardia without signs of acute myocardial ischaemia. Laboratory workup revealed elevated C-reactive protein (40.6 mg/L, ref. <5 mg/L) and leucocytes (12.3/nl, ref. 3.5–9.8/nl). Acute myocardial ischaemia was ruled out by negative troponin test. A CT with contrast-enhancing agent was performed and revealed a peripheral pulmonary embolism, which was however deemed too minor to explain the extent of her symptoms. The CT scan additionally showed a large right atrial (RA) mass measuring 6.2 cm × 3.6 cm, a haemodynamically relevant circular pericardial effusion (4.5 cm) and right-sided mild pleural effusion (1.7 cm) (*Figure 1*). Subsequent transthoracic echocardiography (TTE) confirmed the presence of the mass attached to the lateral and inferior wall of the RA (measuring 5 cm × 3.5 cm) with inhomogenous tissue and smooth borders (*Figure 2*). It appeared to invade the RA myocardium and did not cause obstruction or valvular dysfunction. In order to further characterise the mass, an echocardiographic contrast-enhancing agent (CEA—sulphur hexafluoride microbubbles—SonoVue®, Bracco Imaging, Konstanz, Germany) was utilized, which did not reveal any CEA-uptake of the mass. For further tissue characterization, cardiac magnetic resonance imaging at 1.5 T (Avanto, Siemens Healthineers, Erlangen, Germany) was performed, showing a mass measuring 7.4 cm × 3.6 cm × 7 cm in size in cine images. The tumour occupied the free and inferior wall of the right atrium and invaded the peri- and myocardium. There was a hyperintense signal alteration on T2-weighted STIR imaging at the basal inferior and inferolateral segments in a subepicardial and mid-wall distribution and in parts of the mass, indicative of oedema. Furthermore there was late gadolinium enhancement (LGE) in small parts of the mass and the pericardium. In the perfusion sequences, no contrast enhancement could be demonstrated in the mass and the right atrium (*Figure 3* and Supplementary material online, *Figure 6*).

**Figure 4:**
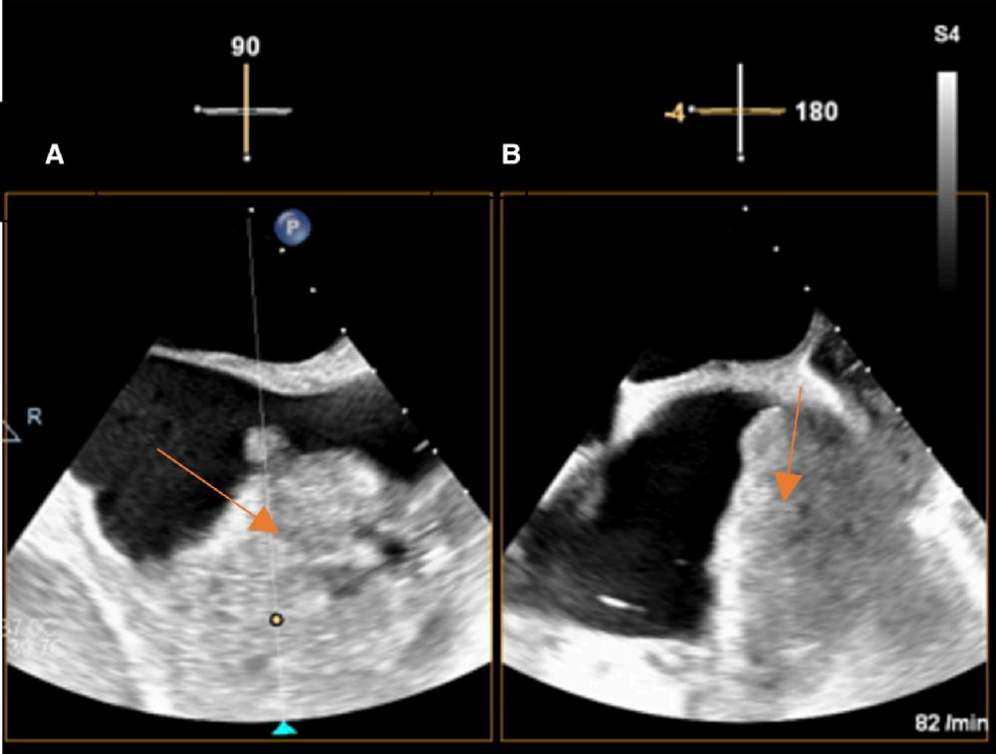
Transoesophageal echocardiogram at 90° in the bicaval view (*A*) and corresponding orthogonal plane (*B*) in the multiplane echocardiogram showing the large mass filling out large parts of the right atrium.

Due its presentation on echocardiography, CT, and MRI, a malignant neoplasm was suspected and conventional transvenous biopsy was attempted in order to obtain tissue samples for histopathological workup. Unfortunately, multiple attempts with different biopsy forceps failed to grasp a sufficient tissue sample—most likely due to a fibrous capsule surrounding the tumour. After interdisciplinary discussion, it was decided to perform a transoesophageal echocardiography (TOE)-guided transvenous Excimer Laser biopsy (*Figure 4* and Supplementary material online, *Figure 5*).

After getting venous access to the right femoral vein (Medtronic Sentrant™, 22F, Dublin, IRL), a guidewire (Amplatz Super Stiff guidewire, Boston Scientific Corporation, Natick, MA, USA) was advanced into the right atrium. Thereafter, we introduced the laser sheath (GlideLight, 80 Hz, 14F, Philips Healthcare, Amsterdam, NL), which was able to penetrate the capsule, by combination of photochemolysis and photothermal ablation.^6,7^

A standard biopsy forceps was then advanced through the laser sheath and was able to recover several tissue samples of different sizes. Histopathological analysis showed atypical cells suspected of malignancy with an epithelioid character. The results were reported to be compatible with an angiosarcoma. After interdisciplinary review, surgery was deemed not feasible, due to infiltration of the pericardium and the large size of the tumour. Therefore, a radiochemotherapy regime was planned, consisting of weekly infusions of paclitaxel (120 mg) combined with stereotactic radiation therapy (60 Gy in 30 fractions). The patient is regularly seen for follow-ups in our oncology outpatient clinic, and the RA mass has reduced in size on TTE, now measuring 1.2 cm × 3.9 cm, 8 weeks after therapy initiation. She currently reports relative freedom from symptoms (*Figure 2C*).

## Discussion

Malignant cardiac tumours are rare but can cause severe morbidity and mortality. In this case report, we demonstrate the importance of multimodality imaging in the clinical workup for cardiac masses. Diagnosing a cardiac tumour can be quite challenging, but recent advances in cardiac imaging have given clinicians the ability to narrow down differential diagnoses before invasive biopsies are performed.^8^ Cardiac CTs are capable of detecting cardiac masses, calcifications, and vascularization depending on the contrast phase and give valuable information on the exact anatomic localization and neighbouring structures. Tumours can be characterized based on their attenuation values (Hounsfield units).^9^ Transthoracic echocardiography and TOE are excellent tools for initial diagnosis, as well as for follow-up scans, due to their combination of excellent image quality with high spatial and temporal resolution and wide availability. The advantages of TTE include non-invasiveness. The TOE has better resolution and both modalities combine the absence of radiation and the ability to measure small anatomical structures. However, limitations include the inability to characterize tissue and patient and/or investigator-dependent variability of image acquisition. Utilizing three-dimensional echocardiography overcomes the limitations associated with geometric assumptions. This approach allows for a more precise evaluation of volume, shape, and the tumour’s relationship to close structures.

Cardiac MRI provides unparalleled non-invasive characterization of cardiac masses due to its uniquely robust tissue characterization and anatomical assessment properties. Typical characteristics of malignant cardiac masses include right heart involvement, myocardial infiltration, pericardial involvement, impaired ventricular wall motion, and heterogeneous T1- and T2-signals pre-contrast, and first-pass perfusion and early and late hyperenhancement post-contrast.^8^

An essential step is the histopathological analysis of a tissue sample. In our case, a traditional biopsy via catheterization did not yield enough tissue, due to the encapsulated nature of the mass, prompting us to perform a TOE- and fluoroscopy-guided biopsy with an excimer laser sheath. Although the sheath was designed for transvenous lead extraction, we could demonstrate its capabilities as a precision bioptome that is able to ensure minimal stress on the surrounding tissue, shallow operational depth, and excellent manoeuvrability.^6,7^

The latest literature indicates that the recommended therapy for angiosarcoma involves surgical resection followed by radiation and chemotherapy with cardiotoxic agents, but most of the patients are inoperable at time of diagnosis. However, there are no standardized treatment algorithms established for this approach. To reduce cardiotoxicity, we switched the chemotherapeutic regimen to paclitaxel, as reported in several case series.^5^ The presented case shows how multimodality imaging and excimer laser-assisted intracardiac biopsy led to the correct diagnosis.

## Supplementary Material

ytae433_Supplementary_Data

## Data Availability

All data underlying this article are available as part of the article. No additional source data are required.

## Lead author biography

Anna Veliqi is a resident physician in the Department of Cardiology and Critical Care Medicine in the Asklepios Klinik St. Georg in Hamburg, Germany. She studied in Hamburg and obtained her degree from the University of Hamburg. Anna Veliqi especially interested in the field of cardiac imaging and structural heart.
